# Progression from on-site to point-of-care fine needle aspiration service: Opportunities and challenges

**DOI:** 10.4103/1742-6413.63195

**Published:** 2010-05-12

**Authors:** Prabodh K Gupta

**Affiliations:** Address: Department of Pathology and Laboratory Medicine, Hospital of the University of Pennsylvania,3400 Spruce St., Pennsylvania – 19104, Philadelphia

**Keywords:** FNA, on-site, point-of-care, Penn-A-Cart, TeleCyP

## Abstract

**Background:**

Standard-of-care requires the availability of an efficient, economical and accurate on-site fine needle aspiration (FNA) service. Presence of a trained individual during the procedure ensures an improved patient care. Appropriate selection of the equipment, interaction with the clinicians and compliance with the various regulations during the procedure is essential. This is often done by an on-site FNA service. Organization and implementation of such a system in a large academic center is challenging.

**Method:**

we reviewed the ambulatory care needs in the new Perelman Center for Advanced Medicine (PeCAM). Multiple (9) FNA sites have been established keeping in view the patient's convenience, clinic demands, various regulatory requirements and laboratory staff. Each location has dedicated FNA station with microscopes and supplies. In addition, state- ofthe -art technologies including a mobile FNA cart (Penn-A- Cart), remote specimen evaluation (TeleCyP) have been incorporated.

**Results:**

The new set up is extremely efficient and much valued by the patients and the clinicians. It has improved patient care.

**Conclusion:**

With necessary investments and resources a point-of-care FNA service has been created which has improved patient care. This, albeit with certain modifications may serve as a model for FNA service.

## INTRODUCTION

After it started as an experimental procedure met with widespread skepticism, fine needle aspiration (FNA) has matured into a vital and dynamic diagnostic tool. This procedure is inherently a team function between the patient, the clinician and the cyto-pathologist. An active dialogue helps ensure diagnostic accuracy in an efficient and economical way. Specimen adequacy and triage, clinicopathologic correlation, and prompt ancillary testing form the linchpins of on-site FNA service and improved patient care. The value of such synergy cannot be over-emphasized. In the context of the current interest in personalized medicine and “transformational pathology”, point-of-care (POC) FNA represents an excellent example of a team approach for delivering personalized diagnostics and care in the most-efficient manner. We briefly present current practices in on-site FNA service and discuss the challenges and opportunities in extending them to a POC model in our institution.

## NEEDLES AND FINE NEEDLE ASPIRATION

The distinctions between needle aspiration, FNA and core needle biopsy, although well known,[1–3] often contribute to confusion. Many a times the terms are used erroneously and interchangeably. While needle aspiration can be performed using 16–20 gauge needles, in the FNA procedures, tissue sampling is done employing a thinner (21–27 gauge) needle, generally attached to a 10 or 20 cc syringe and an aspiration device (handle/gun). Needle aspirations are often performed in dense tissues such as abdominal fat pad and soft tissue lesions. FNA uses the cutting properties of a needle bevel in conjunction with capillary action of the needle shaft to dislodge cells singly and in small clumps or micro-biopsies, preserving cytologic features and relatively few architectural features. Sample extraction and dislodging of tissues is also facilitated by suction applied by the syringe attached to the needle. FNA specimens generally are more concentrated with limited dilution by the fluids and contaminants. Aspirated specimens can be smeared in a number of ways.[4,5] The material obtained by these methods is well-suited for diagnosis. The risk of complications with FNA procedures is minimal. Hematoma may occasionally occur, usually without consequence.[6] There are occasional reports of tumor seeding[7] or fistula formation[8] following an FNA procedure, but it is difficult to prove causality in these rare events.

Core biopsy, on the other hand, is performed with a larger bore needle (10–16 gauges) and yields a larger quantity of tissue requiring histological preparation. This procedure is utilized for predictive and therapeutic studies in lesions of breast, lung, prostate, and other sites with a known or highly suspected malignancy. There is a greater risk for complications including hematoma, cyst and scar formation. Tumor seeding is also a recognized but rare complication of core biopsy.[9–12]

The number of anatomic sites amenable to FNA procedures has grown with improved imaging methods, the development of advanced endoscopic procedures, and greater clinician acceptance.[13–15] There are now relatively few sites that cannot be sampled by fine needle methodologies. Indeed, FNA interpretation facilities preoperative triage of specimens that would not otherwise be amenable to accurate diagnoses. Prior to the development of FNA, pancreatic lesions of nonaggressive radiology were often resected without antecedent tissue diagnoses. As imaging modalities have improved in their ability to detect these lesions, so have the endoscopic methods that make it possible to biopsy them and guide patient management.[16–18]

Localization of the lesion can be immediate, as in the case of a palpable nodule, or may require stepwise positioning of a needle under imaging guidance. Some minimal preparation may be needed for anesthesia or imaging. The time spent for the FNA procedure including on-site evaluation is dependent upon a number of considerations including service proximity, clinical interaction and facilities. The material obtained at each pass is generally smeared, stained and examined for adequacy as well as for interpretation.[2,4,5] Most FNA procedures with on-site evaluation require three or fewer passes per site and in our present set up can be accomplished in 20-40 minutes. Interpretive time is shortened and efficiency is improved by the on-site availability of a cytopathologist. Specimen processing using a number of techniques (on-site smear, cytospin, liquid-based preparation, Millipore filter, and cell blocks) is considerably faster than histological fixation and processing; most specimens are reported in less than 24 hours.

## ON-SITE SERVICE: SPECIAL ADVANTAGES

An on-site interaction between the cytopathologist and the clinician permits opportunity to consult on the history, special issues for consideration, differential diagnosis, specimen triaging and use of ancillary studies. The interdisciplinary approach allows for continual education, understanding, and dialogue that directly benefits patient diagnosis and care.[19]

Patient care is improved when on-site interpretation helps in focusing any additional workup necessary for management. At our institution, FNA specimens with on-site interpretation are routinely triaged for ancillary studies including flow cytometry, molecular testing, microbiology and clinical chemistry (thyroglobulin, PTH, CEA and amylase).[20] This is especially beneficial when the real-time findings are unexpected. On-site assessment permits obtaining additional samples in cases of inadequate or nondiagnostic aspiration, thus increasing overall accuracy. This service also minimizes the number of necessary passes for a given site; in our practice, we rarely (<1%) perform more than three passes.

Thyroid FNA practice at our institution provides a case study in the costs and benefits of on-site service. These aspirations are performed in an interdisciplinary setting and collaboration with endocrinology, radiology and cytopathology departments. In this arrangement, thyroid nodule FNA has attained a 98% diagnostic rate, in concordance with histological diagnosis of 100% for malignant lesions, 67% for those considered suspicious and 56% for those considered indeterminate at FNA.[21,22] The unsatisfactory rate is less than 4% for all FNAs and less than 1% for ultrasound-guided thyroid procedures. In contrast, FNA performed without on-site assessment has a reported nondiagnostic rate of approximately 20%.[22] Based on this data and on average cost per aspiration, it has been estimated that on-site service allows monetary savings on the order of $400,000 per annum at our institution. By examining a broad range of superficial FNAs using 1995 relative value units and reimbursements, Rimm *et al*., calculated savings of $250,000 to $750,000 per 1,000 FNAs performed, or approximately 55,000 RVU.[23]

In many facilities, resources do not always permit an interdisciplinary model; on-site service is often provided by cytotechnologists in a number of facilities. While an experienced cytotechnologist can provide on-site specimen adequacy in simple cases, complex and complicated and unexpected situations require additional professional support. FNA can also be performed by the clinician alone, placing the specimen or prepared slides in an appropriate preservative for delivery to the laboratory. This is sometimes the only practical arrangement. When a cytopathologist is not present for the FNA procedure, the clinician is deprived of an immediate evaluation of specimen adequacy and diagnosis and is forced to defer discussion of the diagnosis and subsequent management to the patient's next visit. Also, the number of suboptimal, unsatisfactory and unnecessary passes and preparations is increased considerably, thus affecting the cost and quality of care. Salient features of the sent-in and on-site FNA procedures are presented in Table 1. There is a distinct need for the laboratory and client interaction to improve patient care.

**Table 1:** Comparison of sent-in and on-site FNA specimens

*Sent-in slides*	*On-site evaluation*
High unsatisfactory rate (>25%)	Low unsatisfactory rate (<2%)
Variable quality of slides	Consistent quality of slides
Slides >20 most cases	slides < 10 most cases
No on-site interpretation, no billing	On-site interpretation, billable
Compromised diagnostic accuracy and patient care	Improved patient management (communication, staging, treatment)
More expensive	Cost effectiveness (reduced number of passes, reduced number of slides, reduced patient in-time).
Inability to triage specimens	Additional testing and triage
Infringement of ACGME requirements	ACGME mandated teaching and training requirements
Minimum investigation potential	Investigational potential
Inability to stay competitive	Standard of care[33]
	Teaching and interactive conferences

Finally, FNA can be performed by the cytopathologist alone. This arrangement although exciting and often advocated has drawbacks. When the clinician is not present at the time of the aspiration, the pathologist besides being deprived of potentially critical information may not sample the right lesion and perform the necessary additional ancillary studies critical for proper patient care. Establishing a separate clinic requires space, personnel, equipment, and regulatory compliance and a cost-benefit analysis. Also, in a large institution it may not always be convenient for the patient to find another location such as an FNA clinic for the procedure.

Challenges for delivery of on-site FNA service include coordination and scheduling, equipment, staff, space, and financial and administrative considerations. The system must be logistically and financially tractable. There is substantial one-time investment in equipment. There are recurrent expenses of the support staff needed for restocking the various supplies and specimen handling. Finally, recent changes in Medicare reimbursement policies for on-site adequacy or interpretation represent additional considerations-and perhaps challenges-for this service model.

### On-Site Service at the Hospital of the University of Pennsylvania 

The Section of Cytopathology and Cytometry at our institution have established a tradition of strong diagnostic services coupled with an active on-site FNA help. The experience of the FNA service at our institution has been one of continuous growth; during the year 2008, nearly 4,000 (3,969) FNA specimens were accessioned in our laboratory. On-site interpretation was provided in over 3,300 (3,353) instances. Routine service is available five days a week during the hours of 8:30 am-5:00 pm and is provided to all clinical areas of the hospital, including inpatient floors, radiology suites, operating rooms, and outpatient clinics in the main hospital and a new, adjacent outpatient (PeCAM) facilities (discussed later). Emergent cases may occur outside of these hours and are covered by the on-call staff.

The on-site FNA service is composed of two teams, each staffed by an attending cytopathologist, a cytopathology fellow and a variable number of pathology residents. Scheduling is handled by two senior secretaries. They gather the patient information and provide bar-coded labels and old lookups to the FNA team. On-site FNA services fall into two categories. In one scenario, the clinician performs the procedure (often under imaging or endoscopic guidance) and cytopathology team prepares the specimen, confirms adequacy, and renders an on-site interpretation.[24] In the second situation, the cytopathologist performs the aspiration, generally for palpable and superficial sites such as subcutaneous nodules, salivary glands, lymph nodes, breast lesions and abdominal fat pads. Either situation can occur in the context of an outpatient or inpatient visit, providing a unique opportunity to raise awareness among patients, their families, and other hospital staff of the key role pathologists plays in the patient's care.

In our main hospital, for on-site evaluations, we use second-generation FNA carts equipped with a dual-headed microscope and necessary supplies.[25] This system, although functional and widely appreciated, is essentially home-brewed to meet clinical needs. The cart is wheeled and parked at various available locations mostly in the patient hallways near an electrical outlet and a sink, representing a safety hazards. Specimens are obtained at the bedside and uncapped needles are carried to the nearby cart generally positioned out side in the crowded hallways. Sensitive information may be discussed in open areas that may violate HIPAA regulations. Procedures are also performed in office and other settings with inadequate infection control precautions.

Building on the on-site service paradigm, we have recently introduced a POC model in our new outpatient facilities. Nearby staff availability, multiple dedicated point-of-care stations, mobile FNA carts, FNA carryalls, available scheduling information, and preprinted labels and pre-numbered slides have allowed us to improve our response time and address the limitations discussed above.

### Point-of-Care Service at the Perelman Center for Advanced Medicine (PeCAM)

**Figure 1:**
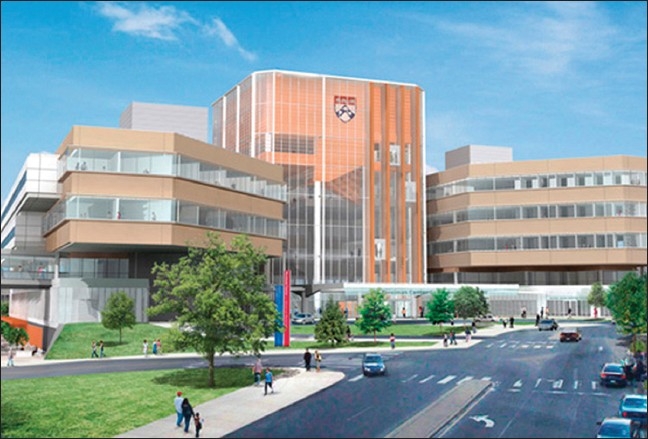
Perelman Center for Advanced Medicine (PeCAM), the new outpatient facility at the University of Pennsylvania. Point-of-care FNA services are provided at various locations throughout the building

The PeCAM at the University of Pennsylvania is a new 500,000 sq ft, five-story, $302 million, state-of-the-art outpatient facility [Figure 1]. In designing an FNA service for this new facility, we adopted the concept of "point-of-care" testing to reflect our commitment to bringing the best FNA practices directly to the bedside.

Challenges in designing point-of-care FNA services included (i) providing efficient service at multiple different locations, currently eight in number; (ii) meeting the Joint Commission on the Accreditation of Health Care Organizations (JCAHO) requirements; (iii) complying with the Health Insurance Portability and Accountability Act (HIPAA) regulations for patient privacy; (iv) complying with infection control regulations; (v) ensuring clinical effectiveness; and finally (vi) meeting the academic mission of the institution.

## FNA SERVICE LOCATIONS

**Figure 2:**
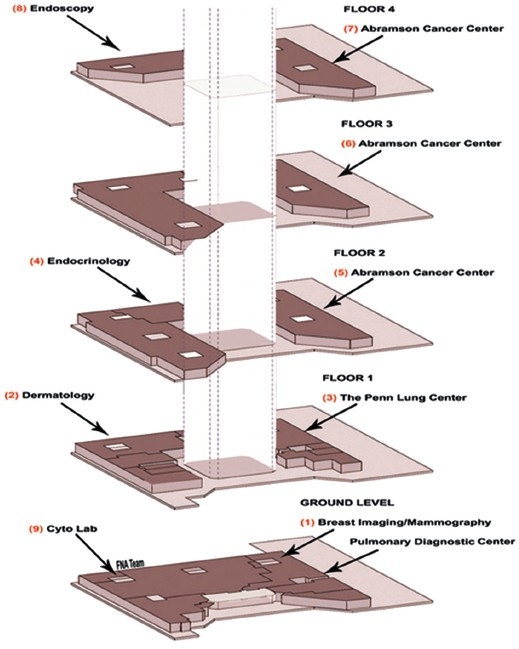
PeCAM floor plans with designated cytopathology service sites.

The PeCAM building is divided into three major wings. In each, patient areas are separated from “white coat” areas. Health care providers have separate sets of elevators and hallways, access is regulated with card keys and trilogy locks. Presently, FNA services are provided at eight service locations [Table 1 and Figure 2]. Each locus is equipped with a dedicated FNA room or station. A cytopathology reading room and associated support facilities are located on the ground floor [#9, Figure 2]. The endoscopy suite [#8, Figure 2] used for bronchoscopy as well as ultrasound guided imaging FNA procedures and cytopathology reading room (#9) are situated in the same core, reducing travel and turnaround time for the care of patients under anesthesia. This layout allows almost all FNA procedures to be completed in less than 15 minutes. While multiple-site sampling, such as in bronchoscopy, may require additional time, in other settings (e.g., thyroid clinic #4) a number of patients can be served sequentially in a short time. In situations with overlapping demands for the FNA service, precedence, is given to the endoscopy patients under anesthesia. Variable number[15–29] of FNA procedures is performed daily; more cases being accessioned on days of the endocrine and follow up clinics.

## EQUIPMENT AND SUPPLIES

Each of the current eight FNA locations is equipped with a microscope permitting multiple simultaneous viewers, a laboratory refrigerator for reagents and specimens, a networked computer, a printer and supplies. Stains and Normsol^®^ in Coplin jars are kept refrigerated, and pre-numbered glass slides, syringes, needles and other items are kept locked at each location. The POC FNA program relies upon the services of a central cytopathology laboratory that is responsible for specimen handling, drop-offs, supplies and restocking. To prevent cross-contamination, each on-site procedure is performed with a fresh set of stains and staining jars.

The design of a multi-site POC FNA program required a significant investment in microscopes and video equipment. High-resolution cameras and screens with live video Axis Camera transmission capabilities are available in endoscopy and the cytopathology reading room (locations #8, #9). A high-definition television is mounted in the mammography station (location #1) for radiograph review. FNA carts of 2^ nd^ and 3^ rd^ generation[25] are used in the endocrinology and lung center locations (#3, #4) and other infrequent sites such as radiation oncology and pulmonary medicine. Multi-headed microscopes are available in the cytopathology reading room (#9). The remaining facilities (#5, #6 and #7) utilize a digital camera and high-definition LCD for multiple viewers. Table 2 summarizes the FNA facilities at these POC service locations. PeCAM facilities although somewhat under used presently, have been designed keeping in view the growth and utilization studies undertaken by the Health System. Additional staffing and TeleCyP will form a major component of the fully functional Center. Superficially, the on-site and POC FNA services appear similar. There are however, subtle differences which provide a more efficient, economical and the state of the art patient care FNA service. Table 3 summarizes the comparison of the two modalities.

**Table 2:** FNA POC service locations at the PeCAM

*Service location [Figure 2]*	*Specimen processing*	*On-site interpretation*
Mammography, ground floor	FNA station	FNA station with high-resolution camera and screen
Dermatology, 2^nd^ floor	Clinic	FNA station with digital camera and screen
Lung center, 1^st^ floor	Clinic, negative pressure room	Penn-A- Cart with digital camera and screen
Endocrinology, 2^nd^ floor	Clinic	Cart with dual-headed scope
Hem/Onc, 2^nd^ floor	Clinic	FNA room with digital camera and screen
Hem/Onc, 3^rd^ floor	Clinic	FNA room with digital camera and screen
Hem/Onc, 4^th^ floor	Clinic	FNA room with digital camera and screen
Endoscopy, 4^th^ floor	Wheeled in cart	FNA room with telepathology facilities
Cytopathology sign-out room, ground floor	N/A	Multiheaded scope with telepathology facilities

**Table 3:** Comparison of on- site and POC FNA service

*Function*	*On-site*	*Point-of-care*
Location sites	Four	Nine
Patient scheduling	No	Yes
Old look-ups	Variable	Yes
Bar labels	Yes	Yes
FNA cart wheeling	Yes	No
Transport wait	Yes	No
Cart set-up	Yes	No
Set-up location	Not designated	Designated
Convenience Power outlet and sink	No	Yes
JACHO compliance	No	Yes
HIPPA compliance	No	Yes
Infectious control compliance	No	Yes
Procedure room location	No	Yes
Direct communication	No	Yes
View of the procedure	No	Yes
Needle localization	No	Yes
Real time accuracy of sampling	No	Yes
Number of passes	Variable	Generally two/three
Specimen triage	Yes	Yes
Cost effectiveness	Less	More
Accuracy Number of passes Reduce # of slides Reduce procedure time Reduce travel and waiting time
Communication (Issues, clinical problems)	Acceptable	Ideal
Teaching and training	Acceptable	Ideal

### The FNA cart

Practical considerations in implementing an on-site FNA service include the need to bring cytopathology equipment and the cytopathologist "into the field". At our institution and at many others, the practice has been to equip a number of wheeled cytology carts with the supplies that are needed to collect, evaluate, and transport specimens. These carts have exhibited varying degrees of technical sophistication over the years,[25] ranging from an open metal cart with two shelves to a "med cart" with increased storage and working space. The most recent prototype in use at our institution (Penn-A-Cart) includes a lift mounted microscope equipped with a camera and LCD display, allowing the entire clinical team to view the specimen along with the cytopathologist and trainees.

An exciting development is the possibility of incorporating TeleCytoPathology (TeleCyP) into the FNA program.[25] By equipping the Penn-A-Cart with a high-speed digital video camera and a computer with wireless internet capabilities, it is possible for an attending pathologist to view glass slides in real time from his or her office, either for primary rendering of a preliminary diagnosis or in consultation. We have piloted this technology at our institution and expect that it will become increasingly common in the future. TeleCyP shall permit FNA services not only within the Health System Network but also remote sites that can be supported by trained support staff members.

### FNA service staffing and workflow

**Figure 3:**
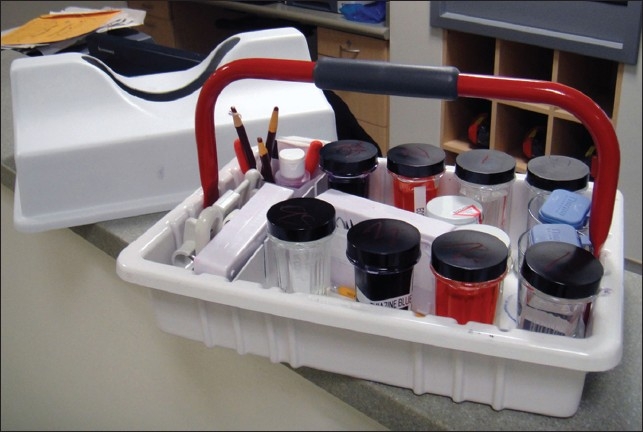
A “carryall” container with a lid used to bring supplies to the bedside. Smears are prepared and taken to an adjacent satellite laboratory for staining and interpretation.

Requests for FNA are received at the front office in the main hospital, the team on call is informed of the location and demographics, look-up documents and bar-coded labels are printed and provided to the team along with the timed request form. For endoscopic and endocrine cases, a list is available in advance and documents are prepared in advance. The FNA team including a cytopathologist, a fellow and a resident if available, is dispatched. An FNA cart or a carryall [Table 4 and Figure 3] containing the essential supplies is brought to the bedside and the procedure is performed. Following on site interpretation, the labeled specimen with documents is double-bagged and returned to the laboratory for further processing.

**Table 4:** Contents of a standard cytopathology carryall

Specimen collection
Syringes (10cc and 20cc), clear hub, with screw or Luer-Lock fittings
Hypodermic needles (#18, 20, 23, 25) in 1.0” and 1½” lengths
Aspiration handle (“gun”) for 10cc and 20cc syringes
Specimen collection buffer (Normosol^®^) in screw-top containers
Alcohol/iodine pledgets, gauze, tape, bandages
Gloves, paper towels, pencils, grease pencils, pens
Blank paperwork (requisition forms, labels, diagnosis reporting forms)
Smear preparation and evaluation
Glass slides, pre-numbered for successive passes (1 through 5)
Diff-Quik stains, 2 sets of 3 Coplin jars
Water container for destaining
Specimen transport
Slide storage box
Ethanol container, leak-proof, for fresh fixed smears

### Patient and staff safety issues

There are several safety concerns in POC FNA practice. Adequate space and illumination are important. The aspiration and phlebotomy procedure's generally involves comparable risks. Precautions must be taken to protect the patient and provider from needle injury, particularly as needles with safety guards cannot be used for FNA procedures. The risk of needle injury increases considerably in endoscopic procedures due to the difficulty of handling the needle catheter. For compliance with the JCAHO regulations regarding safe handling of uncapped needles we prepare smears at the bedside, and then transport them to the FNA area for additional processing and interpretation. Safe needle and biowaste disposal are available on the FNA cart and various locations. FNA specimens are transported in double-walled containers. For compliance with HIPAA regulations, the areas used for FNA interpretation are private and located in close proximity to the procedure location.

### Aerosolization and risk of infection

There is no skin incision in FNA and no propensity to produce airborne droplet nuclei. Reports of tuberculosis transmission from extra-pulmonary sites have generally involved open wounds in known tuberculous patients.[26] We are not aware of any instances of disease transmission attributed to FNA biopsy. On the contrary, FNA has been specifically recommended for the diagnosis of tuberculous lymphadenitis, with reported sensitivity and specificity of 93 and 77 percent.[27–29] There are no specific guidelines of the American Thoracic Society or Infectious Disease Society of America to govern the performance of FNA biopsy.[30–32] It stands to reason, however, that existing infection control policies should be followed when known disease or risk factors are present. In view of the recommendations for infectious (TB) control, all suspected cases of tuberculosis at the PeCAM are processed in negative-pressure environments, which are available at the endoscopic and pulmonary service locations.

## CONCLUSIONS

With careful planning, broad institutional support and committed resources, we have been able to construct a point-of-care FNA service that confirms to current regulations, health care needs and our academic mission, providing an economical, accurate, rapid and goal-directed diagnostic modality. It also offers an opportunity for improved patient care in the future. This paradigm can be adapted to meet the needs of other institutions as a valuable component of contemporary health care.

## DECLARATION OF COMPETING INTERESTS

The authors declare that they have no competing interests.

## AUTHORSHIP STATEMENT BY ALL AUTHORS

Each author acknowledges that this final version was read and approved. All authors of this article declare that we qualify for authorship as defined by ICMJE http://www.icmje.org/#author. Each author has participated sufficiently in the work and take public responsibility for appropriate portions of the content of this article.

## ETHICS STATEMENT BY ALL AUTHORS

As this is case report without identifiers, our institution does not require approval from Institutional Review Board (IRB) (or its equivalent)

## EDITORIAL / PEER-REVIEW STATEMENT

To ensure integrity and highest quality of CytoJournal publications, the review process of this manuscript was conducted under a double blind model (authors are blinded for reviewers and reviewers are blinded for authors) through automatic online system.
